# Microsurgical Reconstruction in Central America: Experience With Free Latissimus Dorsi Flaps at a Public Tertiary Referral Hospital in Costa Rica

**DOI:** 10.7759/cureus.117694

**Published:** 2026-10-09

**Authors:** Joaquín Sánchez-Caballero, Stefanos Koutsowris-Sáenz, Alfredo Sanabria-Castro

**Affiliations:** 1 Plastic Surgery, Hospital San Juan de Dios, Caja Costarricense de Seguro Social, San José, CRI; 2 General Surgery, Hospital San Juan de Dios, Caja Costarricense de Seguro Social, San José, CRI; 3 Research, Hospital San Juan de Dios, Caja Costarricense de Seguro Social, San José, CRI; 4 Hematology and Related Disorders Research Center, Universidad de Costa Rica, San José, CRI

**Keywords:** case series, experience, latissimus dorsi flap, low- and middle-income countries, microsurgical reconstruction

## Abstract

The latissimus dorsi muscle flap is a versatile and dynamic reconstructive option widely used in the management of a variety of oncologic and traumatic injuries and defects. Although microsurgical reconstruction has proven to be highly effective and associated with low complication rates, its implementation in low- and middle-income countries like Costa Rica remains challenging due to technical complexity, limited infrastructure, and a shortage of adequately trained personnel.

This case series presents data from five patients with oncologic or traumatic defects managed by the Plastic Surgery Service at Hospital San Juan de Dios, Costa Rican Social Security Fund (CCSS). Reconstruction of these conditions required coverage using free latissimus dorsi microsurgical flaps. Only one infectious complication was reported, which was successfully treated with medical management without compromising flap viability. Successful functional and aesthetic outcomes were observed in all cases, with results remaining satisfactory throughout five years of follow-up.

## Introduction

Although microsurgical reconstruction with free flaps, including the latissimus dorsi flap (LDF), has transformed the field of reconstructive surgery, its widespread implementation in public hospitals, particularly in developing low- and middle-income countries, remains limited mainly by financial constraints, the requirement of specialized equipment such as surgical microscopes, and the demanding learning curve associated with these procedures [1].

The LDF is widely regarded as a valuable reconstructive option because of its numerous technical advantages. Its vascular anatomy is highly consistent and predictable, featuring a thoracodorsal pedicle of substantial caliber and adequate length, an aspect that facilitates reliable microvascular anastomoses [2]. Previous reports have described anastomosis using surgical loupes when an operating microscope is unavailable, a technique referred to as “macro-microsurgery” [1]. However, all microvascular anastomoses in the present series were performed using an operating microscope. Furthermore, the abundance of cutaneous perforators and the robust vascular supply of the muscle contribute to adequate tissue perfusion [3].

Another important consideration is the relative technical simplicity and versatility of the LDF [4]. Compared with other free flaps, its dissection is generally less complex because of the muscle’s superficial anatomical location and the ease of identifying the thoracodorsal vascular pedicle within the axillary region. These characteristics may contribute to shorter operative times and less demanding training requirements for the surgical team. Supporting this observation, a cross-sectional study conducted at our institution compared the LDF with the transverse rectus abdominis myocutaneous (TRAM) flap [5]. Donor-site morbidity associated with the LDF has also been described in the literature [6]. Overall, these characteristics may make the LDF a practical option in selected resource-constrained settings, depending on local infrastructure, microsurgical expertise, and patient selection. The present series specifically describes free LDF reconstruction of complex non-breast defects.

## Case presentation

A case series describing the main clinical characteristics, surgical management, and outcomes of five patients who underwent microsurgical reconstruction with a free LDF between 2015 and 2020 was conducted. Cases were identified and analyzed retrospectively, and all procedures were performed by the Plastic and Reconstructive Surgery Service and the National Burn Care Unit at Hospital San Juan de Dios, Costa Rican Social Security Fund (CCSS). Clinical data were reviewed to document patient demographics, indications for reconstruction, perioperative course, postoperative complications, and follow-up.

Written informed consent for inclusion in this case series was obtained from all living patients at the time of manuscript development. Case 3 had already passed away from causes unrelated to the reconstructive procedure at the time the manuscript was prepared. A waiver regarding the acquisition of informed consent for this patient was obtained from the Bioethics Department of the Costa Rican Social Security Fund (CCSS). All clinical information was handled in accordance with confidentiality and anonymity standards.

Eligibility criteria

This case series included adult patients (≥18 years), regardless of sex, who presented with soft-tissue defects requiring coverage secondary to oncologic resection or traumatic injury and underwent microsurgical reconstruction using a free LDF.

Patients were excluded if the free LDF was performed exclusively for breast reconstruction. Breast reconstruction was excluded because the present series was designed to evaluate free microsurgical LDF transfer for complex non-breast defects; in our practice, latissimus dorsi breast reconstruction is generally performed as a pedicled rather than a free flap.

Additional exclusion criteria included severe or decompensated systemic disease that substantially increased anesthetic, surgical, or microvascular risk; radiotherapy within 12 months before reconstruction; pregnancy; or active infection at the recipient site at the time of definitive reconstruction. Controlled conditions such as hypertension or diabetes, without significant end-organ dysfunction or clinical decompensation, were not considered major comorbidities or exclusion criteria.

A total of five clinical cases were included in this series. The defects involved two main anatomical regions: scalp/frontal region (three cases) and lower extremities (two cases). Each case was assessed according to the reconstructive indication, surgical technique, microsurgical anastomoses, postoperative complications, and clinical outcomes during follow-up.

A previous episode of infection or sepsis that had been adequately treated and clinically controlled before flap transfer was not considered an active infection at the time of reconstruction.

In all cases, the LDF was selected because of its reliable vascular anatomy, ability to provide a large volume of well-vascularized tissue (skin paddle), resistance to infection, and versatility for transfer to distant recipient sites via microsurgical techniques, even in challenging settings such as post-traumatic defects and extensive oncologic resections [7,8]. Furthermore, the thoracodorsal pedicle, which generally has a diameter that exceeds 2 mm, facilitates safe and reproducible microvascular anastomoses. These established anatomical and technical advantages were important considerations in selecting both the flap and the recipient vessels [9].

For the cephalic reconstructions, particular consideration was given to the length of the vascular pedicle and its ability to reach distant recipient sites while allowing adequate flap rotation. This anatomical configuration has been described as a reliable option for complex head-and-neck defects [10].

In lower‑limb cases, the LDF was selected when regional reconstructive options were unsuitable because of extensive traumatic tissue loss or failure of previous reconstruction attempts, consistent with current guidelines for limb reconstruction [11].

Surgical technique

Prior to surgery, all patients underwent a standardized preoperative assessment according to the institutional protocol of the Plastic Surgery Department at Hospital San Juan de Dios. Prophylactic antibiotic therapy consisted of 2 g of intravenous vancomycin administered one hour before the procedure, with an additional intraoperative dose given at the time of vascular anastomosis. Alternative regimens, including oxacillin or clindamycin, were used when indicated according to allergy history and individual clinical considerations.

During the procedure, the latissimus dorsi muscle was meticulously dissected while preserving its vascular pedicle. A cutaneous paddle was incorporated when required to provide additional soft-tissue coverage, facilitate three-dimensional reconstruction, or address specific functional needs of the injury or defect.

Recipient vessels were selected intraoperatively according to the location of the defect, vessel availability, and characteristics of the surgical bed. In the head and scalp reconstructions, the superficial temporal vessels were used in case 1 and the facial artery and vein in cases 2 and 3. In the below-knee stump reconstruction, a genicular branch of the popliteal vessels was used, while the posterior tibial vessels were used in the heel reconstruction.

All arterial and venous microvascular anastomoses in the five cases were performed using an operating microscope.

The mean operative time was 8.3 hours, with procedures ranging from eight to nine hours depending on injury complexity and the condition of the recipient site. The median length of hospital stay was 10 days, ranging from five to 15 days (Table 1). Variations in hospitalization were primarily related to the extent of the injury, the patients' overall clinical condition, and postoperative recovery course. Follow-up ranged from one to five years.

**Table 1:** Summary of cases. LDF: latissimus dorsi flap; SSI: surgical Site Infection; y: years; M: male; F: female. Data provided by the Unified Digital Health Record (EDUS), Costa Rican Social Security Fund, Costa Rica.

Case	Age (y)	Sex	Indication	Location and recipient vessels	Size (cm)	Discharge time	Complications	Follow-up
1	35	M	Trauma	Frontoparietal scalp. Superficial temporal vessels	12 x 8	9 days	Postoperative *P. aeruginosa* SSI with partial flap necrosis in the scalp	Annually for 5 years post surgery
2	68	F	Cancer	Vertex scalp. Facial artery and vein	16 x 10	12 days	None	Annually for 5 years post surgery
3	60	M	Cancer	Frontal scalp/cranial. Facial artery and vein	14 x 10	5 days	None	Annually for 2 years post surgery
4	38	M	Trauma	Below-knee stump, right leg. Genicular branch of popliteal vessels	16 x 8	10 days	None	Annually for 5 years post surgery
5	30	M	Trauma	Left heel. Posterior tibial vessels	8 x 6	15 days	None after LDF flap.	One year after final reconstruction

Scalp and head frontal region reconstruction

Case 1

A 35‑year‑old male with no significant past medical history sustained severe frontoparietal trauma and bilateral upper-extremity avulsion injuries following a traffic accident. Upon admission, the patient was in critical condition and required multidisciplinary management. His clinical course was complicated by sepsis, necessitating intensive medical treatment and prolonged hospitalization. The sepsis was treated, and the patient was clinically stabilized before microsurgical reconstruction.

Initial management included multiple surgical procedures (irrigation and debridement) involving both soft tissue and bone in the frontal region. Because of the extent of the defect, reconstruction was performed using a free LDF with microvascular anastomosis to the superficial temporal vessels. A 12 × 8 cm skin paddle was incorporated to provide adequate coverage of the frontal defect. The patient remained hospitalized under the care of the Orthopedic Service because of his associated upper-extremity injuries. During the reconstruction postoperative period, the patient developed a surgical-site infection. One of the most common complications of cranioplasty, reported in 5% to 26% of cases [12]. The causative organism, *Pseudomonas aeruginosa*, was isolated and treated with IV meropenem 2 g every eight hours for 10 days, with close monitoring of renal function. The infection was associated with partial flap necrosis, requiring a scalp rotation flap to achieve definitive wound closure. No total free-flap loss occurred (Figures 1-4).

**Figure 1:**
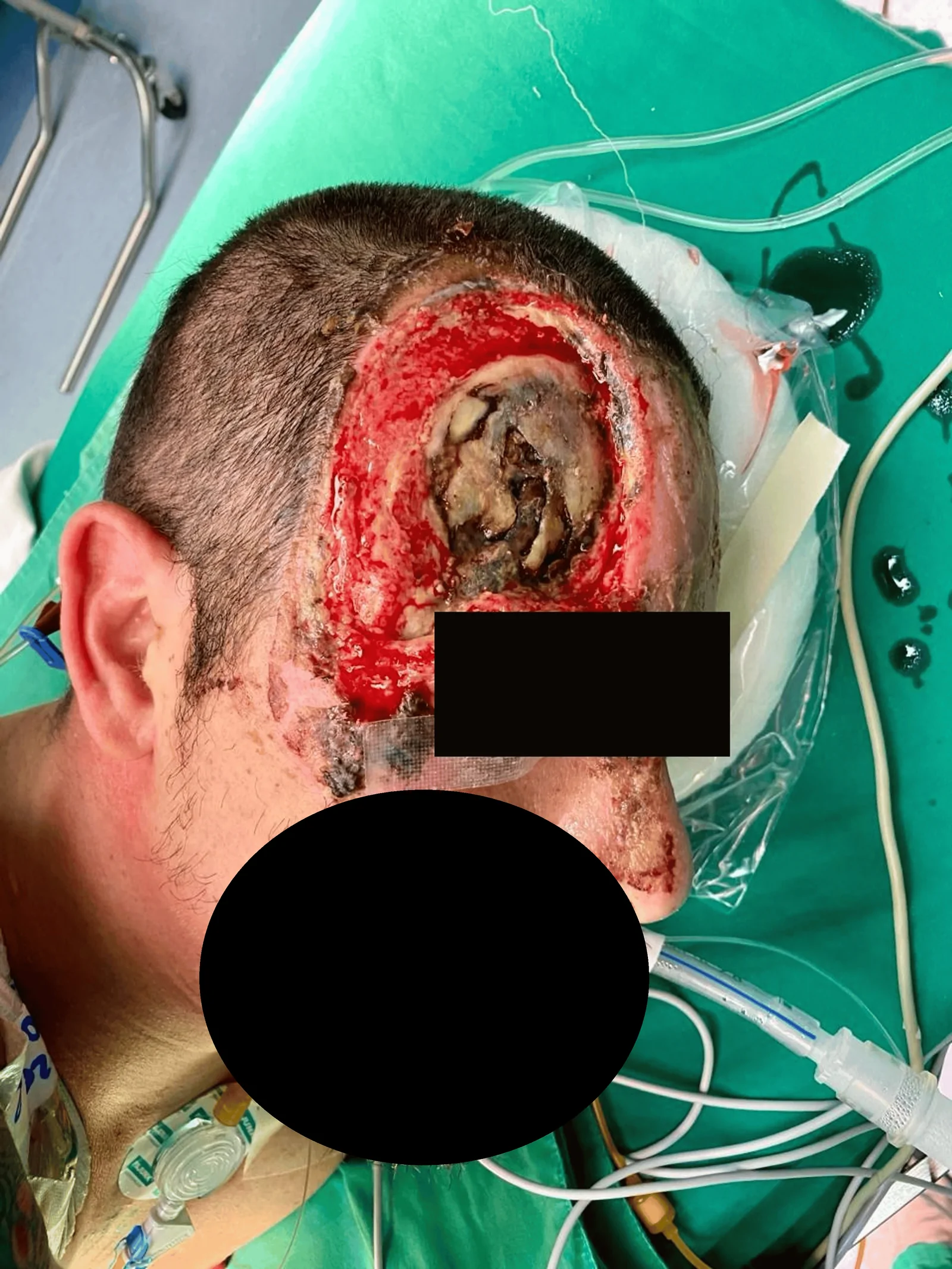
Patient with an open traumatic lesion prior to reconstruction.

**Figure 2:**
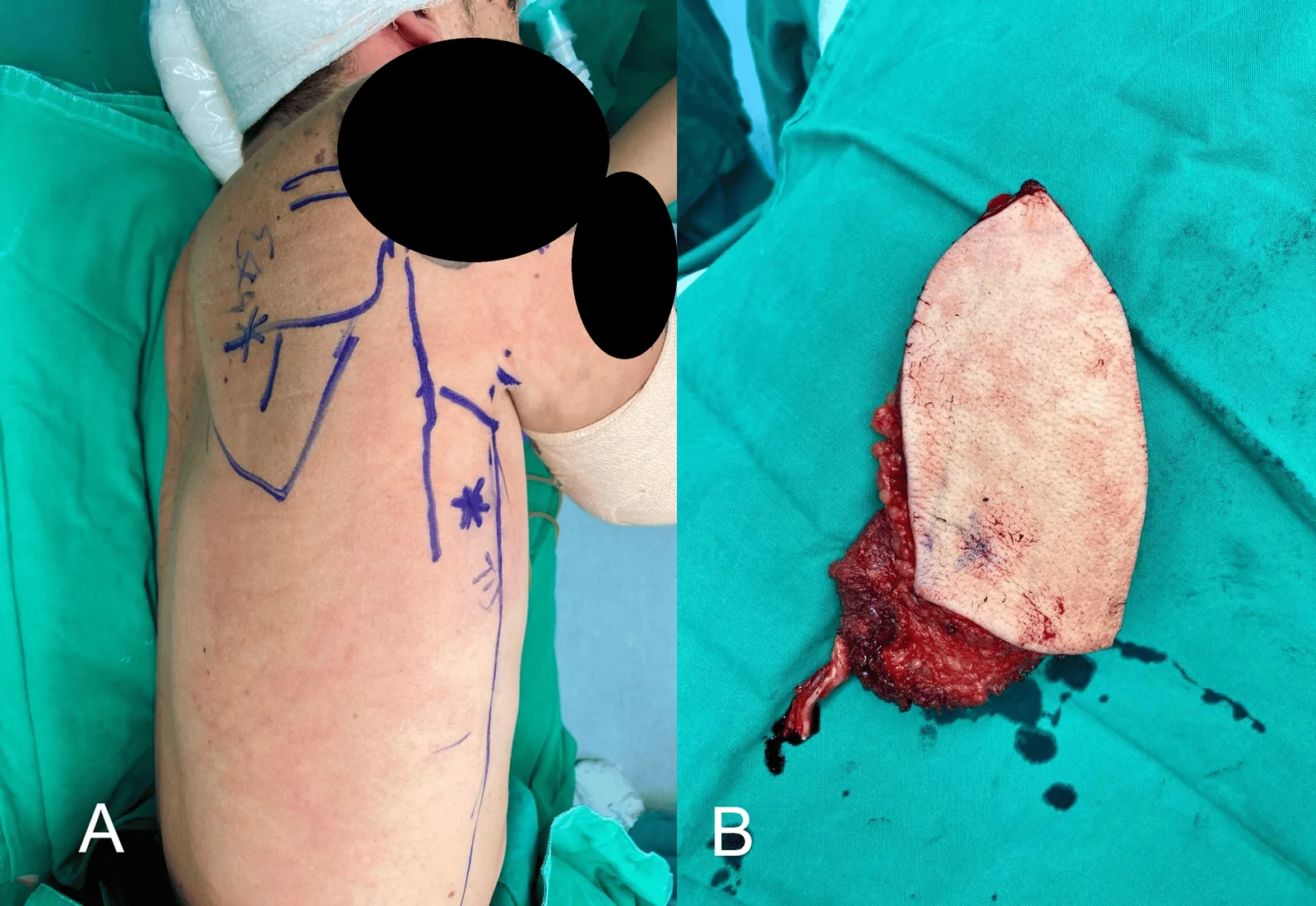
Surgical planning prior to reconstruction. A: Delineation. B: Extraction of the latissimus dorsi flap.

**Figure 3:**
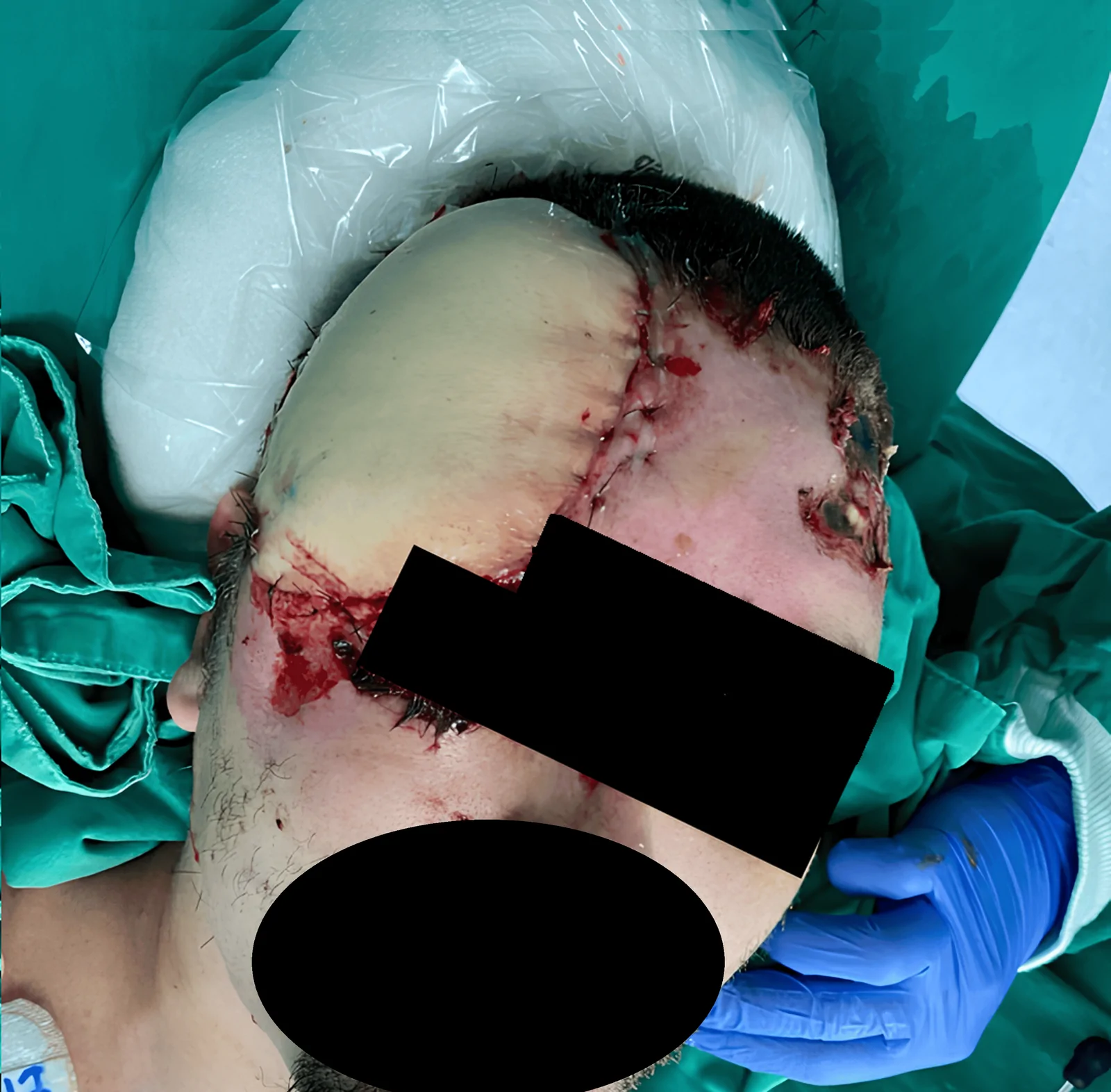
Patient after latissimus dorsi flap microsurgical reconstruction.

**Figure 4:**
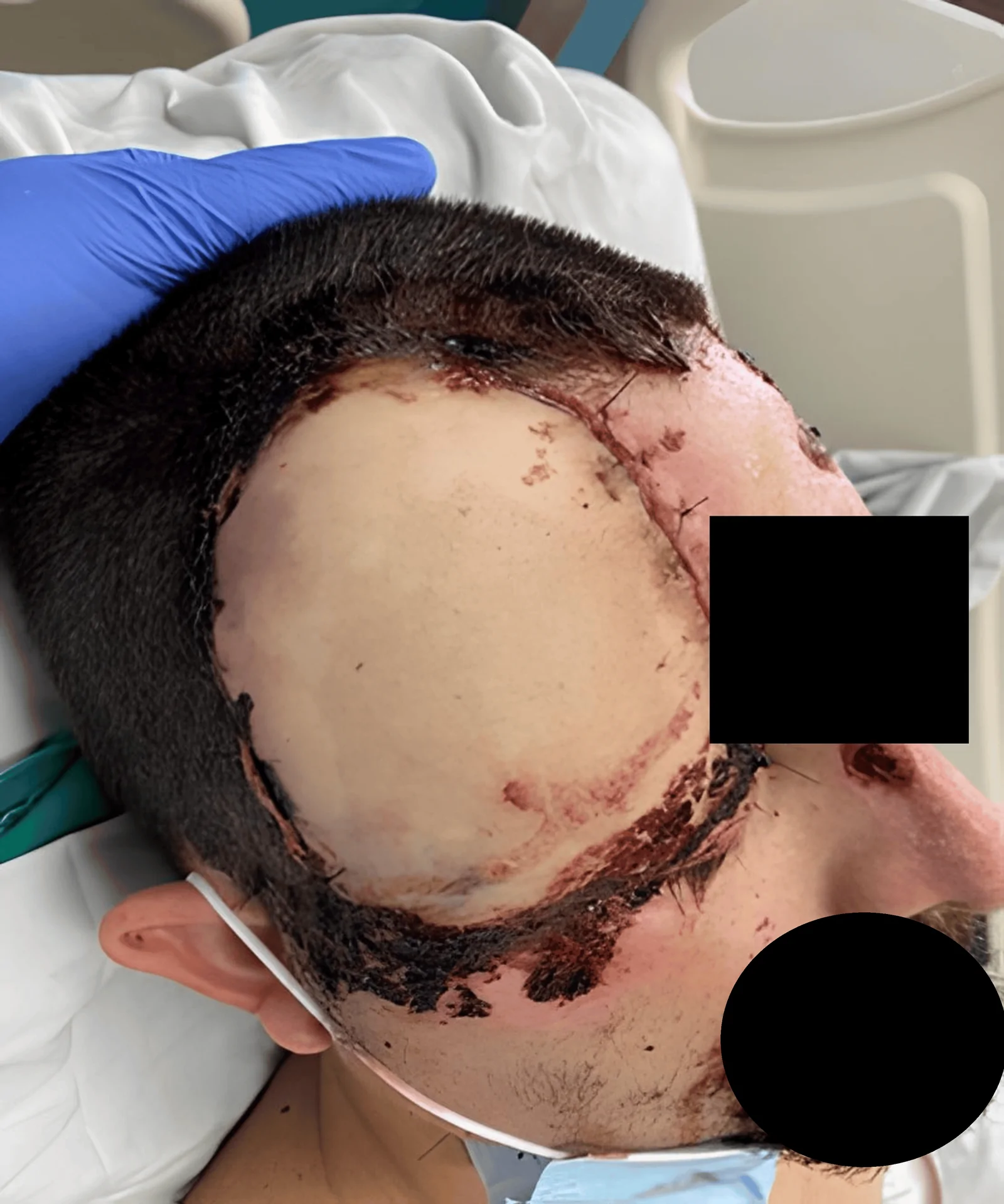
Patient after latissimus dorsi flap microsurgical reconstruction.

Long-term follow-up was maintained for five years, during which no complications were observed.

Case 2

A 68-year-old female with a history of controlled hypertension underwent successful resection of a vertex scalp sarcoma with oncologically clear margins (Figure 5). Reconstruction was performed using a free LDF featuring a 16 × 10 cm skin island, along with a skin graft placed over the lateral surface of the muscle. The facial artery and vein were used as recipient vessels. The postoperative course was stable and without complications, allowing for discharge after a 12-day hospital stay. Over a five-year follow-up period, she remained free of complications and demonstrated satisfactory clinical progression (Figure 6).

**Figure 5:**
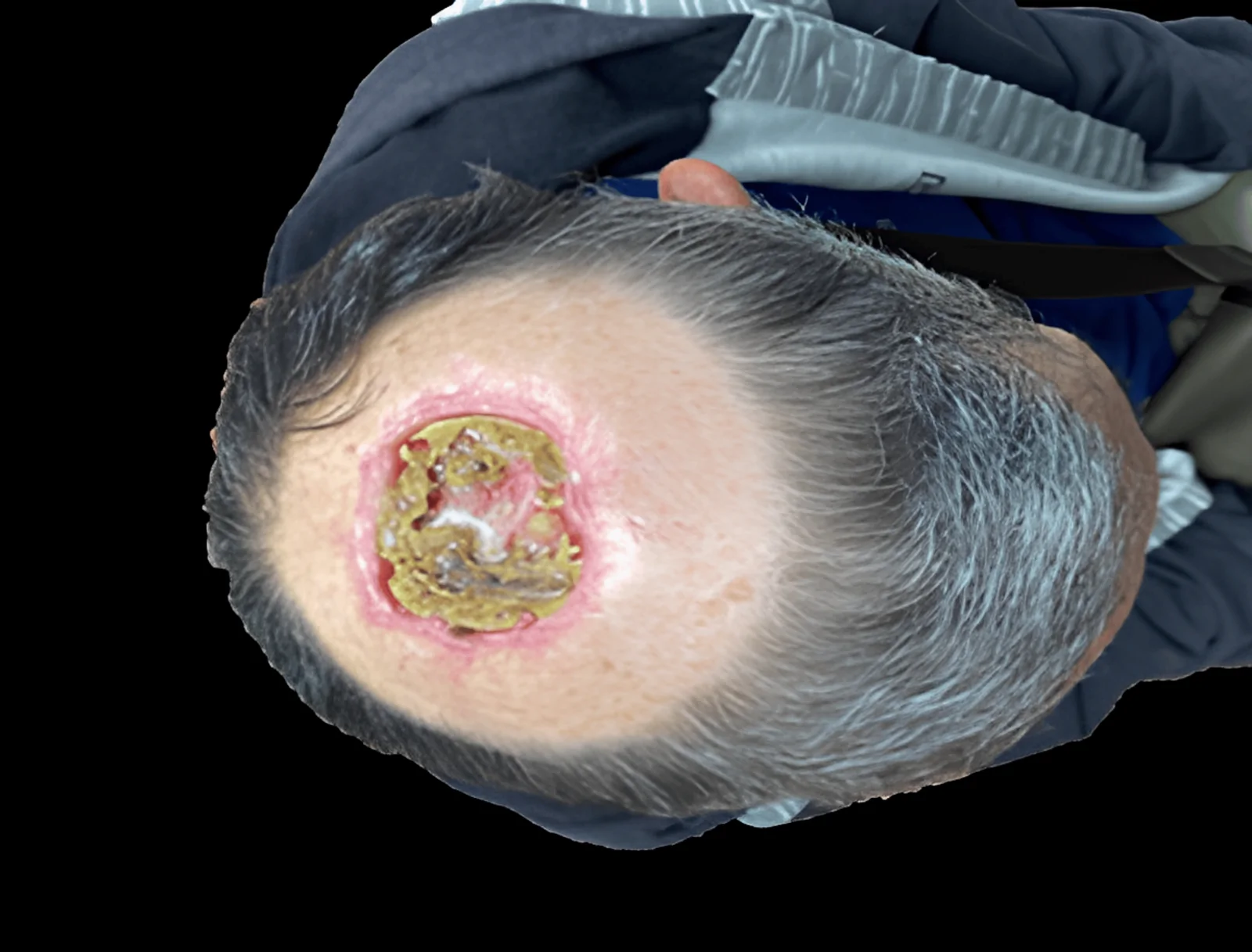
Patient with vertex sarcoma prior to reconstruction.

**Figure 6:**
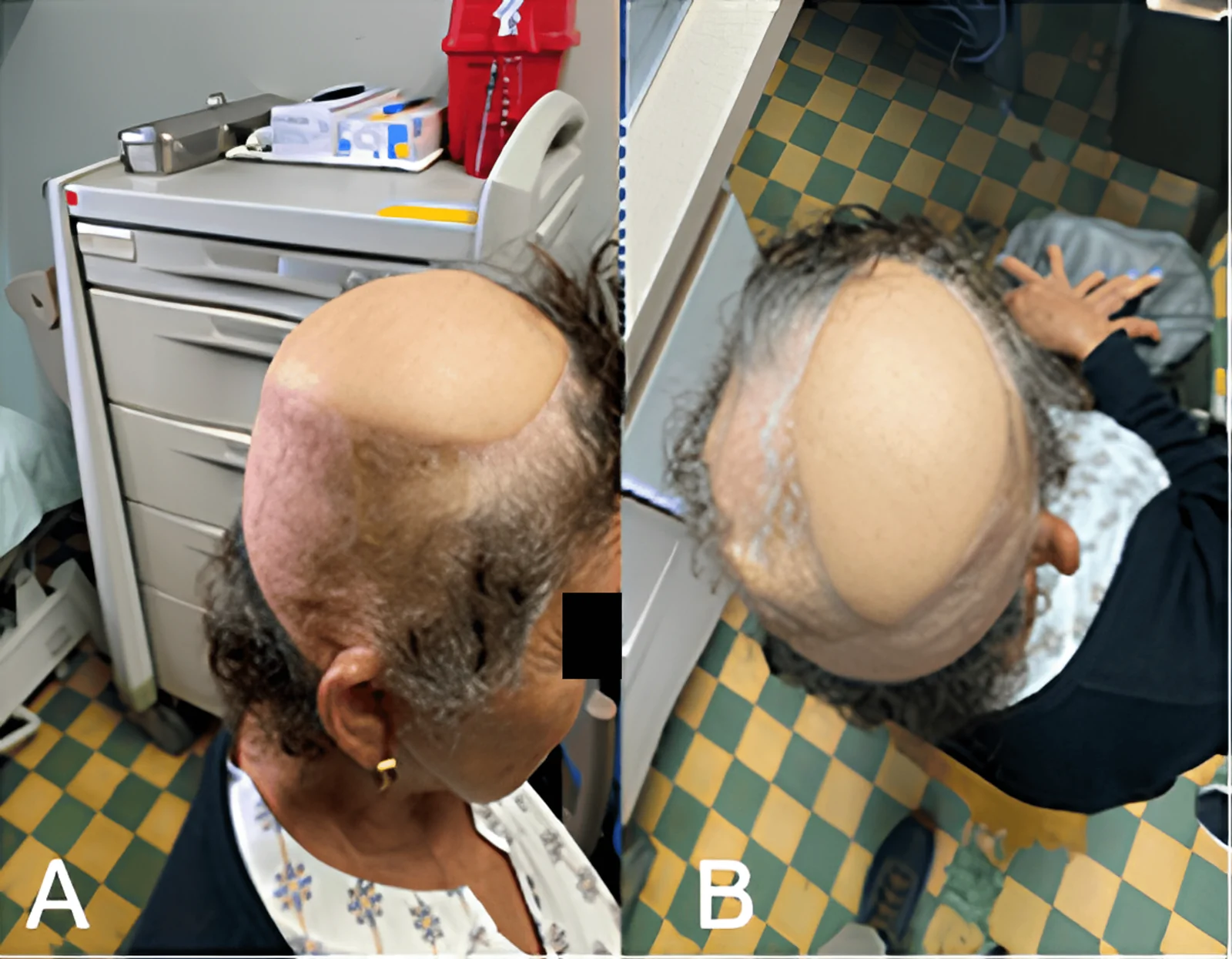
Patient with a history of vertex sarcoma after latissimus dorsi flap microsurgical reconstruction. A: Lateral view. B: Posterior view.

Case 3

A 60-year-old male with a significant smoking history (approximately two packs per day for more than 20 years) was diagnosed with invasive squamous cell carcinoma of the frontal scalp with underlying calvarial involvement. Initial management included a multidisciplinary radical resection of the scalp, involved soft tissues, and frontal bone. The dura mater was also affected, resulting in a large composite defect that required reconstruction with a titanium mesh and screw fixation system. Definitive soft-tissue coverage was subsequently achieved through microsurgical reconstruction using a free LDF of approximately 14 x 10 cm, with the facial artery and vein used as recipient vessels. The postoperative course was favorable, and the patient was discharged on postoperative day five. Follow-up was available for two years. The patient subsequently died from causes unrelated to the surgical intervention (Figures 7-10).

**Figure 7:**
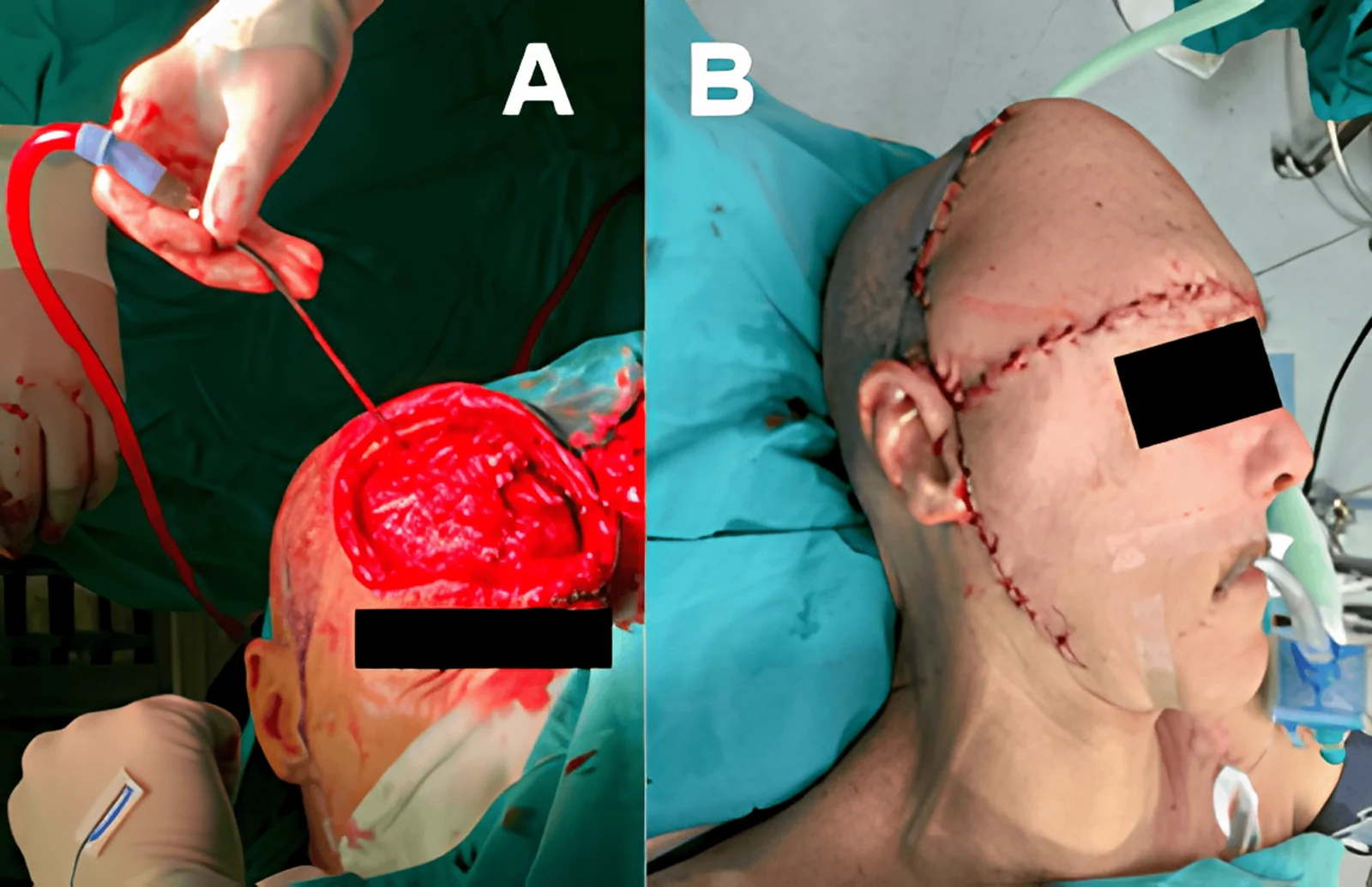
Patient with a history of invasive squamous cell carcinoma during reconstruction surgery. A: Previous surgery. B: After reconstruction.

**Figure 8:**
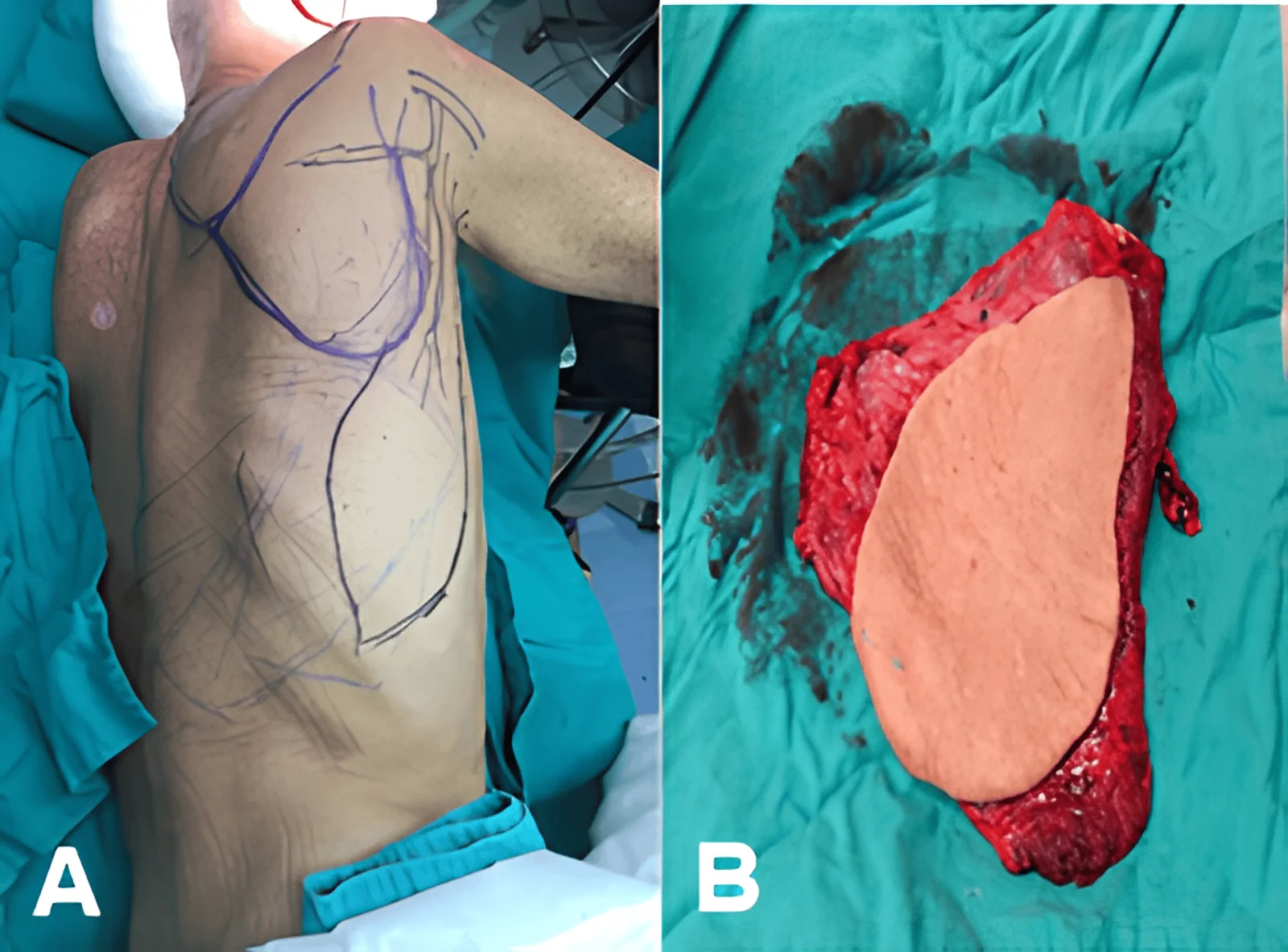
Surgical planning prior to reconstruction. A: Delineation. B: Extraction of the latissimus dorsi flap.

**Figure 9:**
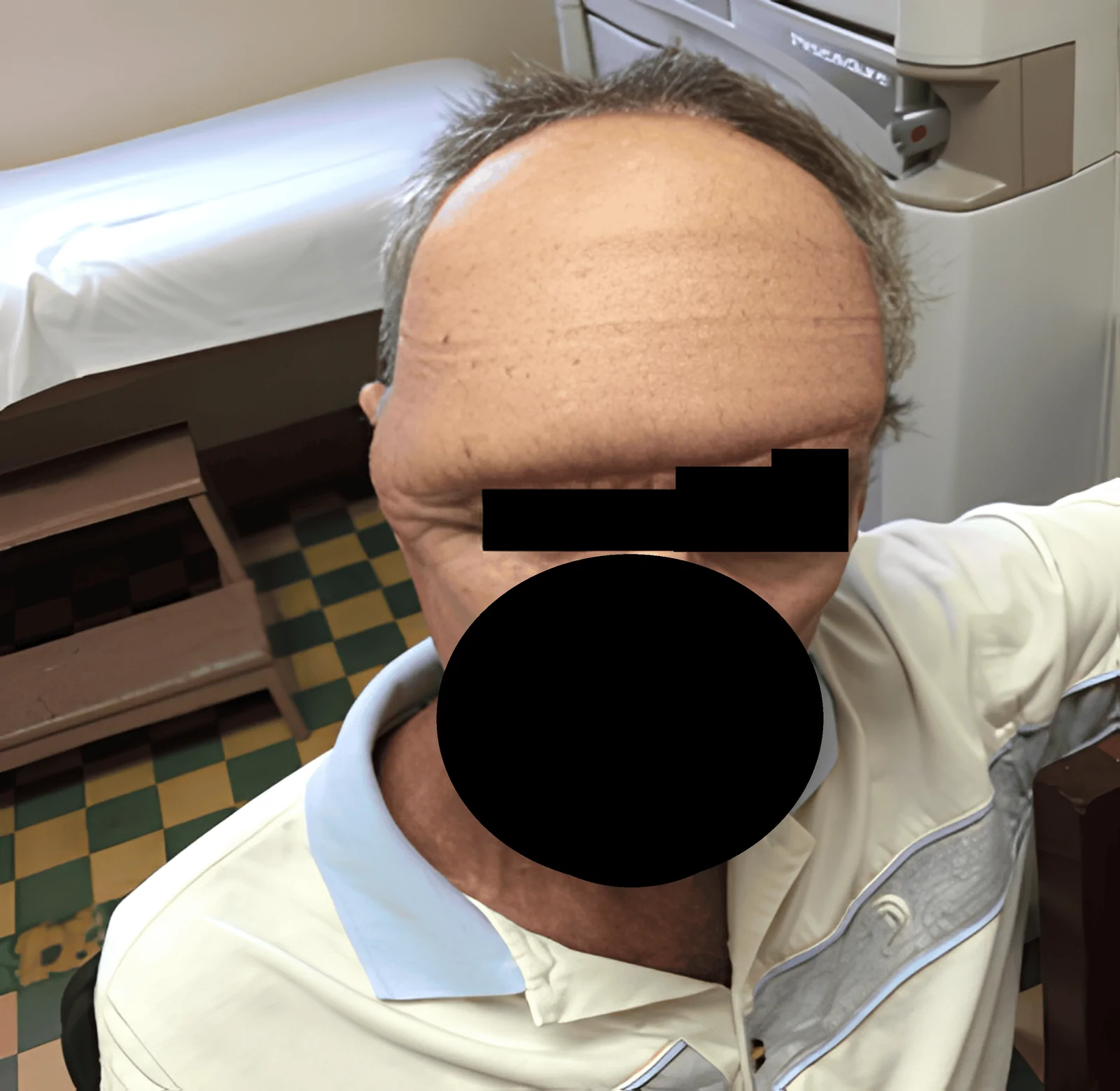
Patient after latissimus dorsi flap microsurgical reconstruction.

**Figure 10:**
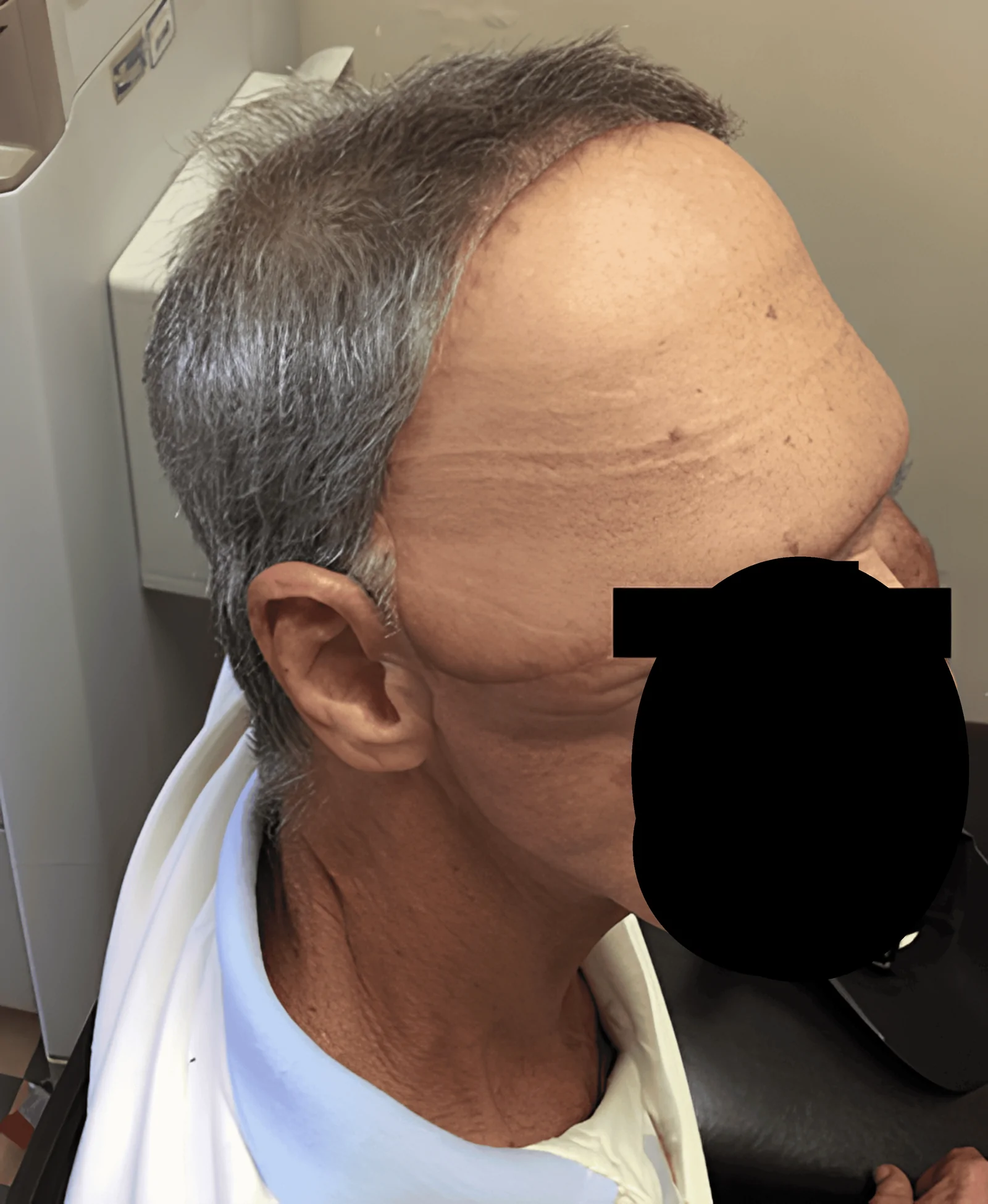
Patient after latissimus dorsi flap microsurgical reconstruction.

Lower‑limb reconstruction

Reconstruction of defects of the distal lower extremity remains particularly challenging because of the limited availability of reliable local and regional flap options in this anatomical region [13,14]. Common reconstructive alternatives include perforator-based flaps, such as the reverse sural flap, dorsalis pedis flap, lateral supramalleolar flap, and flaps based on perforators from the peroneal or posterior tibial vessels [15]. However, the utility of these techniques may be significantly restricted following high-energy trauma, where local donor tissues are frequently compromised or unavailable.

In such circumstances, LDF represents a versatile and dependable reconstructive alternative. The flap can be harvested with or without a skin paddle and tailored to the dimensions and requirements of the defect, allowing coverage of extensive areas while maintaining a low rate of major complications. Its adaptability also facilitates reconstruction in anatomically complex regions. Based on these considerations, the free LDF was selected for reconstruction in the following clinical cases [15].

Case 4

A 38-year-old male polytrauma patient underwent a right below-knee amputation following a motorcycle accident. Six months after the event, he developed recurrent pressure ulcers at the amputation stump associated with prosthetic use. Initial conservative management, including prosthesis replacement and adjustment, failed to resolve the condition. Consequently, microsurgical reconstruction with a free LDF was indicated to provide durable soft-tissue coverage while minimizing excessive tissue bulk and preserving prosthetic function.

A 16 × 8 cm skin paddle was designed over the area corresponding to the most prominent portion of the stump. The flap was successfully transferred and revascularized through microvascular anastomosis to the genicular branch of the popliteal vessels. The flap demonstrated satisfactory integration, with no early or late postoperative complications during the 10-day hospital stay. During follow-up, no recurrence of pressure ulcers or other major flap-related complications was documented (Figure 11).

**Figure 11:**
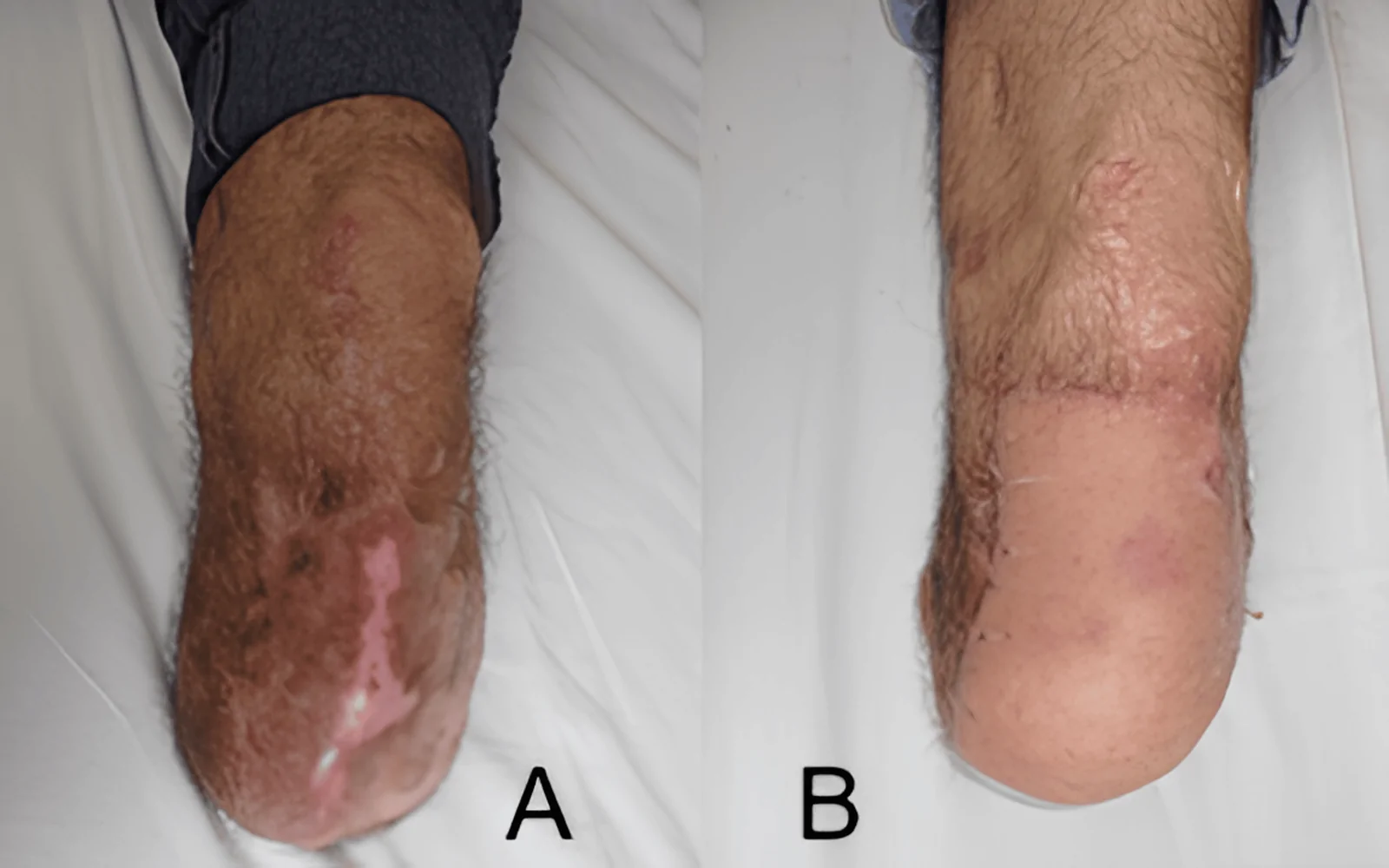
Lower‑limb reconstruction. A: Exposed stump. B: Stump covered with a latissimus dorsi flap.

Case 5

A 30-year-old male with insulin-dependent diabetes mellitus sustained a motorcycle-related injury resulting in extensive soft-tissue loss involving the left heel. Initial management included surgical debridement, followed by an attempt at reconstruction with a reverse sural flap performed by the Orthopedic Service. However, the flap developed vascular thrombosis and subsequent necrosis within three days of surgery, requiring its removal. Following repeat debridement, definitive reconstruction was performed using a free LDF, and wound coverage was completed with a meshed split-thickness skin graft. The posterior tibial vessels were used as recipient vessels. This approach provided durable soft tissue coverage of the calcaneal region and facilitated successful limb preservation (Figures 12-15).

**Figure 12:**
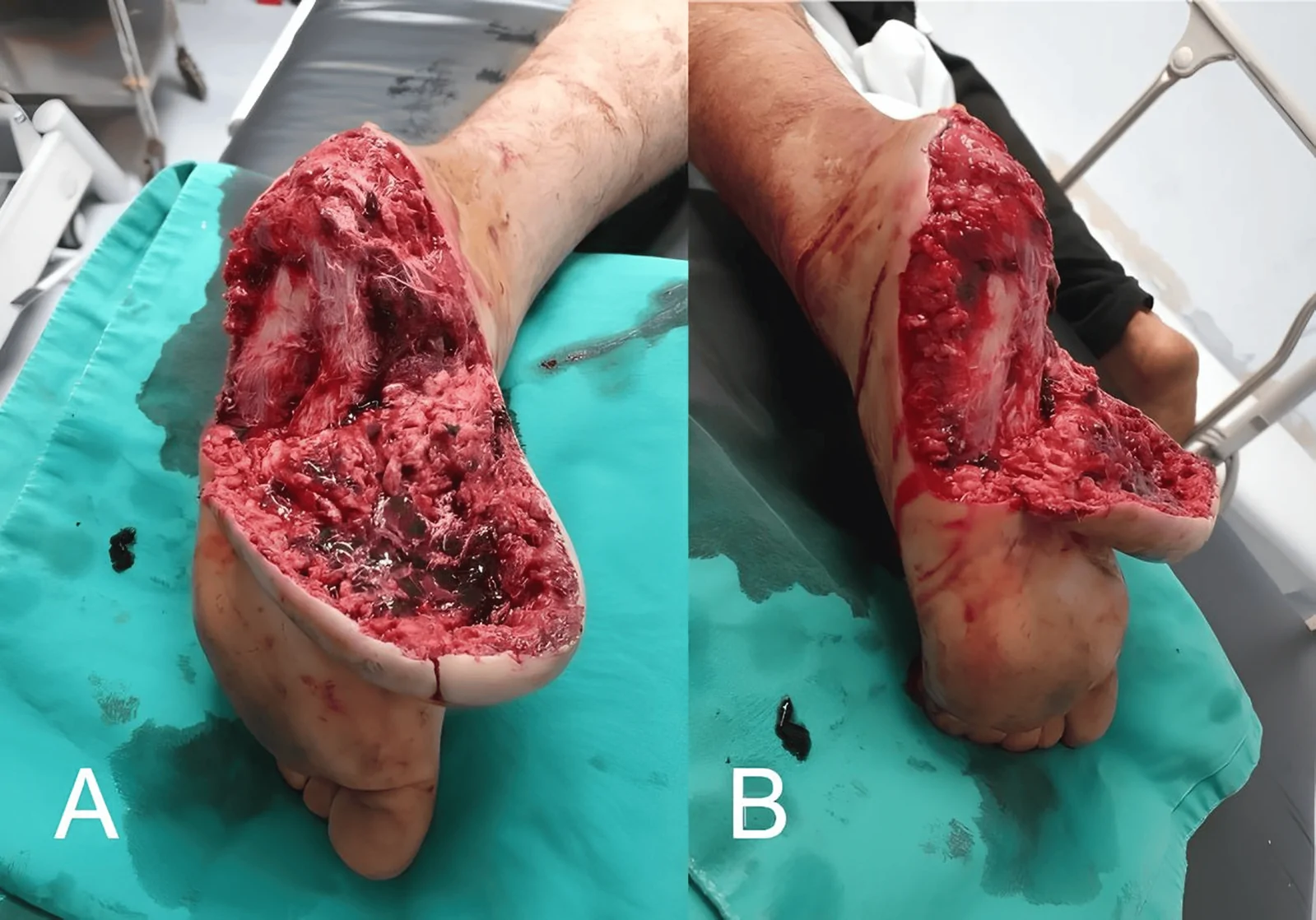
Initial lesion of the foot. A: Posterior view. B: Lateral view.

**Figure 13:**
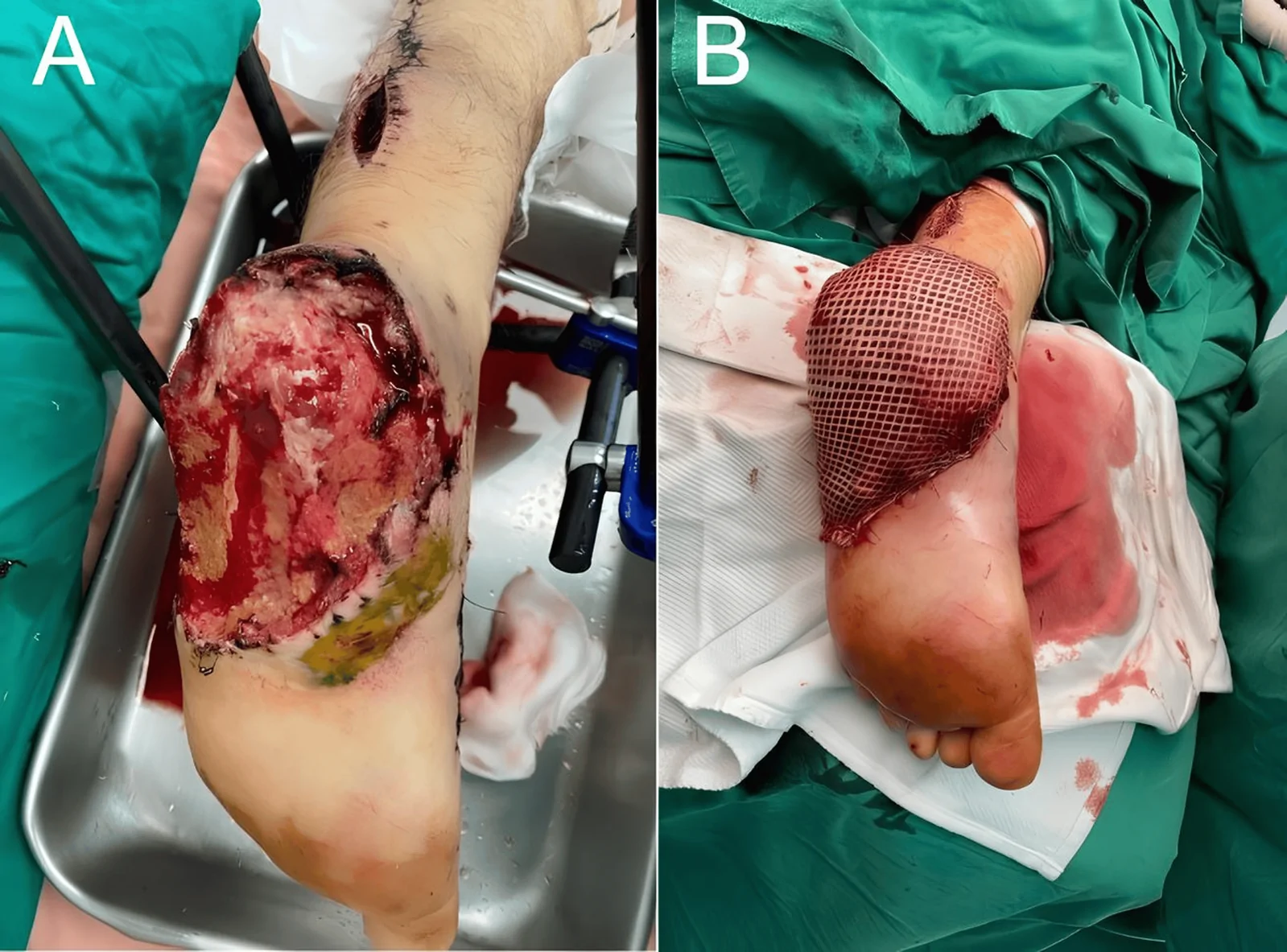
Reconstruction process. A: Removal of the reverse sural flap. B: Free latissimus dorsi flap with a meshed split-thickness skin graft.

**Figure 14:**
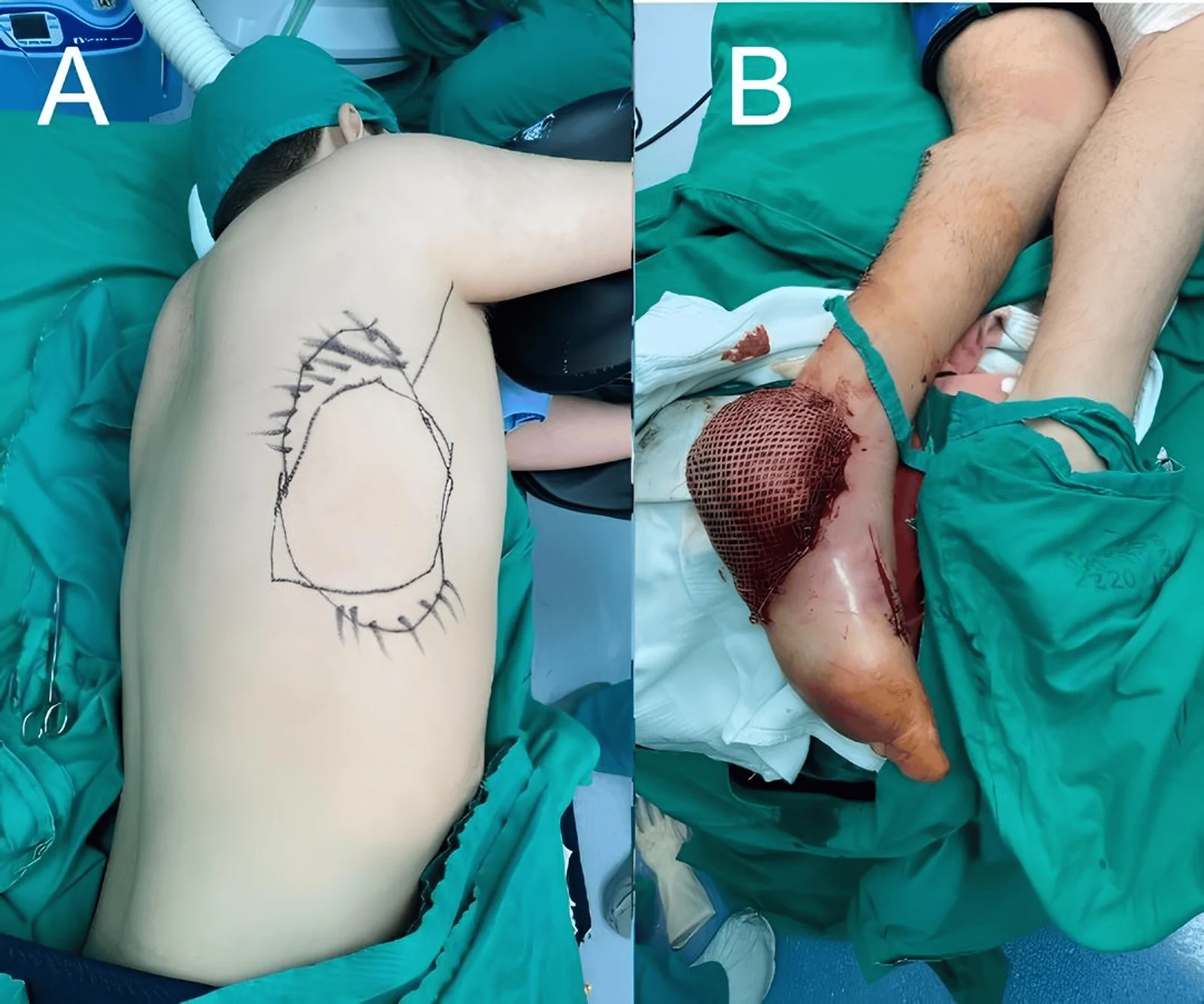
Reconstruction process. A: Delineation. B: Heel after final reconstruction.

**Figure 15:**
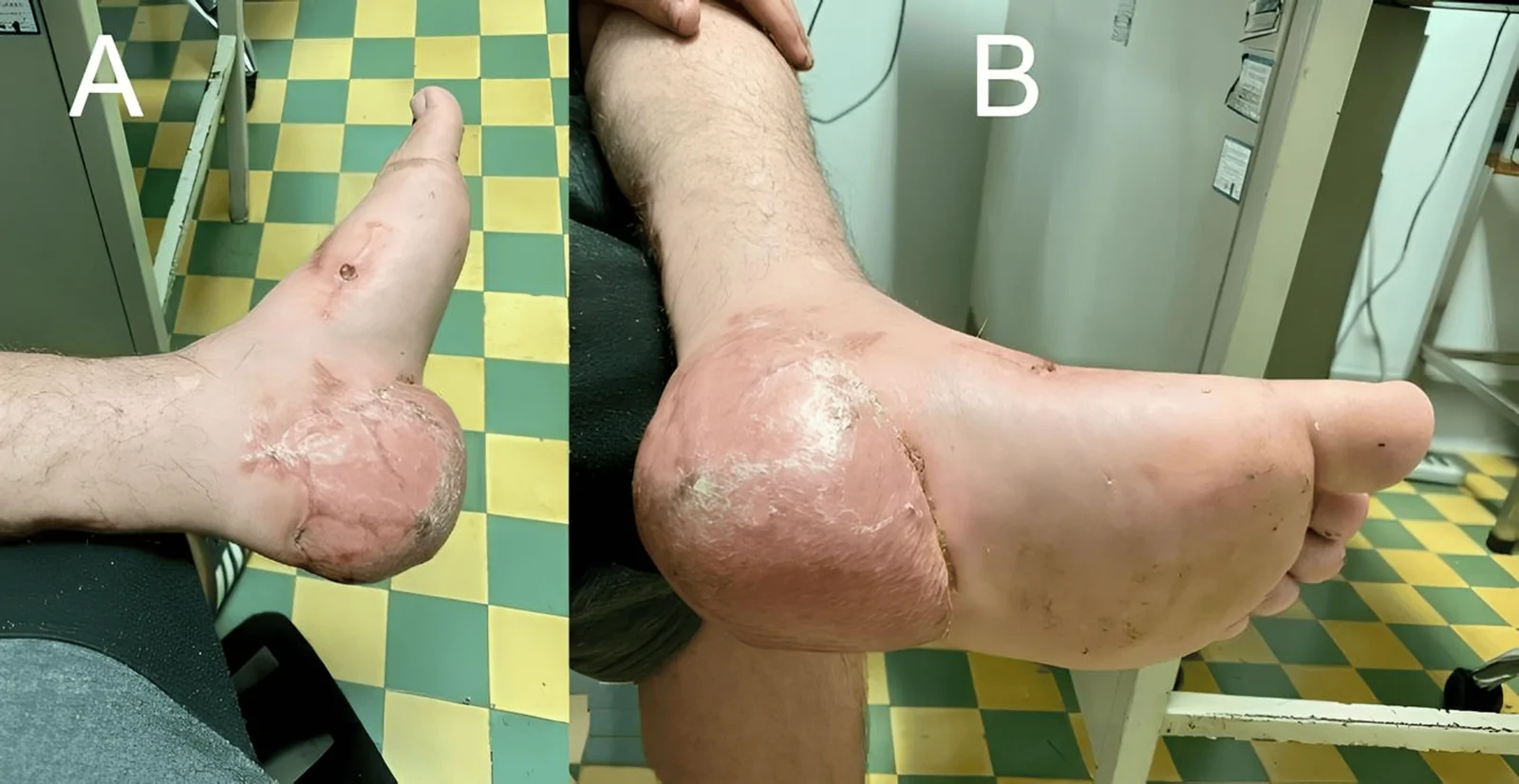
Patient with a history of foot trauma after latissimus dorsi flap microsurgical reconstruction. A: Lateral view. B: Front view.

One year after the final reconstruction, the flap remained integrated without major LDF-related complications.

Findings across cases

Throughout the study period (2015-2020), at least five patients underwent micro‑surgical reconstruction with a free LDF in the Plastic and Reconstructive Surgery Unit at Hospital San Juan de Dios.

Surgical indications were primarily related to defects resulting from oncologic resections and high-energy traumatic injuries, involving anatomical regions such as the scalp and lower extremities. Trauma-related defects occurred in younger patients, all of whom were male, whereas reconstructions following oncologic resection were performed in older individuals (Table 1). All five free flaps survived without total flap loss. One patient (20%) developed a postoperative *Pseudomonas aeruginosa* surgical-site infection associated with partial flap necrosis and required an additional scalp rotation flap for definitive wound closure. No other major LDF-related complications were documented during the available follow-up. 

In several procedures, the flap was combined with additional reconstructive materials, including titanium meshes, silicone implants, and biocompatible spacers, to restore osseous integrity or provide three-dimensional structural support in complex defects, particularly those involving the cranial vault and amputation stumps.

Among the five cases analyzed, one patient (20%) developed a postoperative surgical-site infection that was successfully managed with targeted antimicrobial therapy, without compromise of flap viability (Table 1).

No other major complications were observed in the remaining patients during hospitalization or throughout the follow-up period. Furthermore, no flap losses, functional limitations, or unfavorable aesthetic outcomes were reported, and all patients achieved satisfactory reconstructive results (key factors to be considered in cases undergoing this procedure).

Pedicled LDFs were also employed for other reconstructive procedures at our unit, including the upper extremity, chest wall, neck, and breast reconstruction, with favorable clinical and functional outcomes. These cases were excluded from the present case series because no informed consent was obtained from patients or the procedure did not involve microsurgical free tissue transfer.

## Discussion

The management of oncologic and polytraumatized patients with extensive soft-tissue defects represents a substantial component of the workload of the Plastic, Reconstructive and Aesthetic Surgery Unit at Hospital San Juan de Dios (CCSS). Such defects frequently pose significant reconstructive challenges, particularly when local or regional flap options are inadequate to provide durable coverage because of the distal location of the defect, exposure of critical structures, or extensive tissue loss.

In our experience, the latissimus dorsi free flap has emerged as a versatile and reliable option for complex reconstruction. Its adaptability permits application across a broad spectrum of clinical scenarios [4,15]. According to the specific reconstructive needs, the flap may be harvested as a musculocutaneous flap with a skin paddle or as a muscle-only flap. It can also be combined effectively with adjunctive reconstructive materials, including titanium meshes and silicone prostheses, when restoration of skeletal contour or structural support is necessary. These characteristics are relevant to the management of complex soft-tissue defects across multiple anatomical regions.

In our series, postoperative complications consisted of one surgical-site infection associated with partial flap necrosis, without total flap loss. This case is relevant to reports of latissimus dorsi free-flap reconstruction in infected cranial defects [12]. For lower-extremity defects, free-tissue transfer remains an established option when local or regional tissues are unavailable or previous reconstruction has failed [7,8,13-15]. Breast reconstruction literature was retained only as general background regarding donor-site morbidity and long-term function, because breast reconstruction was not included in this case series [16,17].

Functional and aesthetic outcomes were not assessed using standardized or validated instruments in this retrospective series. Therefore, these outcomes are described only clinically and should not be interpreted as formal evidence of functional or aesthetic superiority.

The main limitations of this study are the small sample size, retrospective design, heterogeneous indications, absence of a comparison group, and variable follow-up ranging from one to five years. These limitations restrict the generalizability of the findings.

In our experience, the latissimus dorsi flap has frequently represented a useful reconstructive option because of the location and extent of the defects treated. Its well-established anatomy and technical versatility may be advantageous in selected patients. In resource-constrained public hospitals, its practical value depends on available microsurgical expertise, operating-room resources, and appropriate patient selection.

## Conclusions

In this retrospective series of five selected patients, microsurgical reconstruction using a free LDF provided durable soft-tissue coverage for complex traumatic and oncologic defects of the scalp/cranial region and lower extremity. No total flap loss occurred; however, one patient developed a postoperative *Pseudomonas aeruginosa* surgical-site infection with partial flap necrosis that required additional local reconstruction. These findings represent our institutional experience and support the feasibility and versatility of the free LDF in selected cases. Because of the small sample size, retrospective design, absence of a comparison group, and variable follow-up, broader conclusions regarding safety, superiority, cost-effectiveness, or first-line use cannot be established from this series.
